# Chemokine receptor–targeted PET/CT provides superior diagnostic performance in newly diagnosed marginal zone lymphoma patients: a head-to-head comparison with [^18^F]FDG

**DOI:** 10.1007/s00259-023-06489-6

**Published:** 2023-11-09

**Authors:** Aleksander Kosmala, Johannes Duell, Simone Schneid, Sebastian E. Serfling, Takahiro Higuchi, Alexander Weich, Constantin Lapa, Philipp E. Hartrampf, Markus Raderer, Hermann Einsele, Andreas K. Buck, Max S. Topp, Wiebke Schlötelburg, Rudolf A. Werner

**Affiliations:** 1 Department of Nuclear Medicine, University Hospital Würzburg, Oberdürrbacher Strasse 6, 97080 Wurzburg, Germany; 2 Department of Internal Medicine II, University Hospital Würzburg, Wurzburg, Germany; 3 Faculty of Medicine, Dentistry and Pharmaceutical Sciences, Okayama University, Okayama, Japan; 4 Nuclear Medicine, Faculty of Medicine, University of Augsburg, Augsburg, Germany; 5 Department of Internal Medicine I, Medical University Vienna, Vienna, Austria; 6 Department of Radiology and Radiological Sciences, Johns Hopkins School of Medicine, Baltimore, MD USA

**Keywords:** [^18^F]FDG, CXCR4, C-X-C motif chemokine receptor, PET, [^68^Ga]Ga-PentixaFor, Marginal zone lymphoma

## Abstract

**Background:**

In patients with marginal zone lymphoma (MZL), [^18^F]FDG PET/CT provided inconsistent diagnostic accuracy. C-X-C motif chemokine receptor 4 (CXCR4) is overexpressed in MZL and thus, may emerge as novel theranostic target. We aimed to evaluate the diagnostic performance of CXCR4-targeting [^68^Ga]Ga-PentixaFor when compared to [^18^F]FDG PET/CT in MZL.

**Methods:**

Thirty-two untreated MZL patients (nodal, *n* = 17; extranodal, *n* = 13; splenic, *n* = 2) received [^68^Ga]Ga-PentixaFor and [^18^F]FDG PET/CT within median 2 days. We performed a visual and quantitative analysis of the total lymphoma volume by measuring maximum/peak standardized uptake values (SUV_max/peak_), and calculating target-to-background ratios (TBR, defined as lesion-based SUV_peak_ divided by SUV_mean_ from blood pool). Visual comparisons for both radiotracers were carried out for all target lesions (TL), and quantitative analysis of concordant TL evident on both scans. Last, MZL subtype analyses were also conducted.

**Results:**

On a patient-based level, [^68^Ga]Ga-PentixaFor identified MZL manifestations in 32 (100%) subjects (vs. [^18^F]FDG, 25/32 [78.1%]). Of the 256 identified TL, 127/256 (49.6%) manifestations were evident only on CXCR4-directed imaging, while only 7/256 (2.7%) were identified on [^18^F]FDG but missed by [^68^Ga]Ga-PentixaFor. In the remaining 122/256 (47.7%) concordant TL, [^68^Ga]Ga-PentixaFor consistently provided increased metrics when compared to [^18^F]FDG: SUV_max_**,** 10.3 (range, 2.53–37.2) vs. 5.72 (2.32–37.0); SUV_peak_, 6.23 (1.58–25.7) vs. 3.87 (1.54–27.7); *P* < 0.01, respectively. Concordant TL TBR on [^68^Ga]Ga-PentixaFor (median, 3.85; range, 1.05–16.0) was also approximately 1.8-fold higher relative to [^18^F]FDG (median, 2.08; range, 0.81–28.8; *P* < 0.01). Those findings on image contrast, however, were driven by nodal MZL (*P* < 0.01), and just missed significance for extranodal MZL (*P* = 0.06).

**Conclusions:**

In newly diagnosed MZL patients, [^68^Ga]Ga-PentixaFor identified more sites of disease when compared to [^18^F]FDG, irrespective of MZL subtype. Quantitative PET parameters including TBR were also higher on [^68^Ga]Ga-PentixaFor PET/CT, suggesting improved diagnostic read-out using chemokine receptor-targeted imaging.

## Introduction

Representing approximately 7% of all indolent non-Hodgkin’s lymphomas, marginal zone lymphomas (MZL) comprised three distinct subtypes: extranodal MZL (EMZL) of mucosa-associated lymphoid tissue, nodal MZL (NMZL), and splenic MZL (SMZL) [1, 2]. For initial workup of newly diagnosed MZL, current practice guidelines recommend contrast-enhanced computed tomography (CT) as imaging modality of choice [3]. The value of 2-deoxy-2-[^18^F]fluoro-D-glucose ([^18^F]FDG) positron emission tomography/CT (PET/CT) for routine initial staging is a matter of debate, with reported variable radiotracer accumulation depending on MZL subtype, or other histopathologic and morphologic features [4–9]. Nonetheless, respective guidelines recommend the use of this PET agent in challenging scenarios, e.g., for guidance of biopsies or treatment monitoring of localized treatment [3].

Beyond [^18^F]FDG assessing glucose consumption in sites of disease, PET agents specifically targeting other (sub)cellular structures overexpressed in MZL have been evaluated in recent years [10, 11]. For instance, the C-X-C motif chemokine receptor 4 (CXCR4)-directed radiotracer [^68^Ga]Ga-PentixaFor showed improved read-out capabilities when compared to guideline-compatible work-up, including CT. Those previous results, however, did not focus on a comprehensive head-to-head comparison of [^68^Ga]Ga-PentixaFor vs [^18^F]FDG in all lymphoma manifestations. As such, we aimed to compare findings on both PET/CTs in untreated MZL patients, including a visual and quantitative assessment on a patient and target lesion (TL)-level specifically focusing on the whole body lymphoma load.

## Methods

We performed a retrospective analysis of our institutional PET/CT database and identified 32 newly diagnosed MZL patients who received [^68^Ga]Ga-PentixaFor and [^18^F]FDG PET/CT within no more than 30 days. Patients did not receive oncological treatment between PET scans. All patients provided written informed consent. The local institutional review board waived the need for additional approval as this was a retrospective investigation (waiver no. 20220414 01). Prior studies have reported on parts of this patient cohort [10, 12, 13]. In [10], only a “hottest lesion” analysis was conducted; i.e., exclusively, the most intense lesion on PET has been further investigated, while in the present study, a lesion-based head-to-head comparison of the entire lymphoma burden has been performed.

### Imaging procedure

PET/CT scans were obtained on Biograph mCT64 or 128 scanners (Siemens Healthineers, Erlangen, Germany). The scans covered an area from the top of the head to the upper thigh. For [^18^F]FDG scans, a minimum fasting period of 6 h was required, to ensure a blood glucose level below 180 mg/dl. For both radiotracers, imaging was initiated one hour after injection, with a median injected activity of 115 MBq (range, 78–186) for [^68^Ga]Ga-PentixaFor scans, and 299 MBq (range, 239–406) for [^18^F]FDG scans. Image acquisition in 3D mode was performed with a flow bed velocity of 1.1 mm/s (mCT128) or 2 min/bed position (mCT64). The images were reconstructed by applying a Gaussian filter of 2.0 mm with a 200 × 200 matrix in three iterations (subsets, 24/21 [mCT 64/128]). Attenuation correction was based on CT scans with or without contrast enhancement [10, 14].

### Image analysis

A dedicated workstation and software package (syngo.via, version VB60A; Siemens Healthineers, Erlangen, Germany) were used to evaluate images. For a readout of the entire lymphoma load, one reader (S. Sc.) placed spherical volumes of interest (VOIs) around all potential target lesions (TL) with tracer uptake above background levels. Another reader (A. K.) verified all VOIs, and inconclusive findings were reviewed in consensus with two expert readers (S. E. S., R. A. W.). All readers were blinded to clinical data and other imaging results, including the respective other PET scan. After having placed a VOI, the software automatically generated a three-dimensional contour at a 40%-threshold, thereby providing maximum/peak standardized uptake values (SUV_max_, SUV_peak_). For target-to-background-ratio (TBR) calculations, SUV_peak(TL)_ was divided by the averaged mean blood pool SUV from three regions of interest on separate slices in the vena cava superior [10, 12]. For a head-to-head comparison of both radiotracers, two readers (S. Sc., A. K.) identified TL that were identical on both PET scans.

### Statistical analysis

We used the GraphPad Prism version 9.3.1 (GraphPad Prism Software, La Jolla, CA, USA) for statistical analyses. Continuous normally distributed (as per Shapiro-Wilk test) variables are presented as mean ± standard deviation; otherwise, median and range are provided. To compare concordant TL, we applied the Wilcoxon matched-pairs signed rank test. For other group comparisons, the Mann-Whitney test was used.

*P* < 0.05 was considered statistically significant.

## Results

Mean patient age was 65.6 ± 13.4 years. 19/32 (59.4%) patients were female, and 13/32 (40.6%) were male. Distribution among subtypes based on molecular imaging findings was as follows: 17/32 (53.1%) NMZL, 13/32 (40.6%) EMZL, and 2/32 (6.3%) SMZL.

### Total lymphoma load on CXCR4-directed and [^18^F]FDG PET/CT

**Fig. 1:**
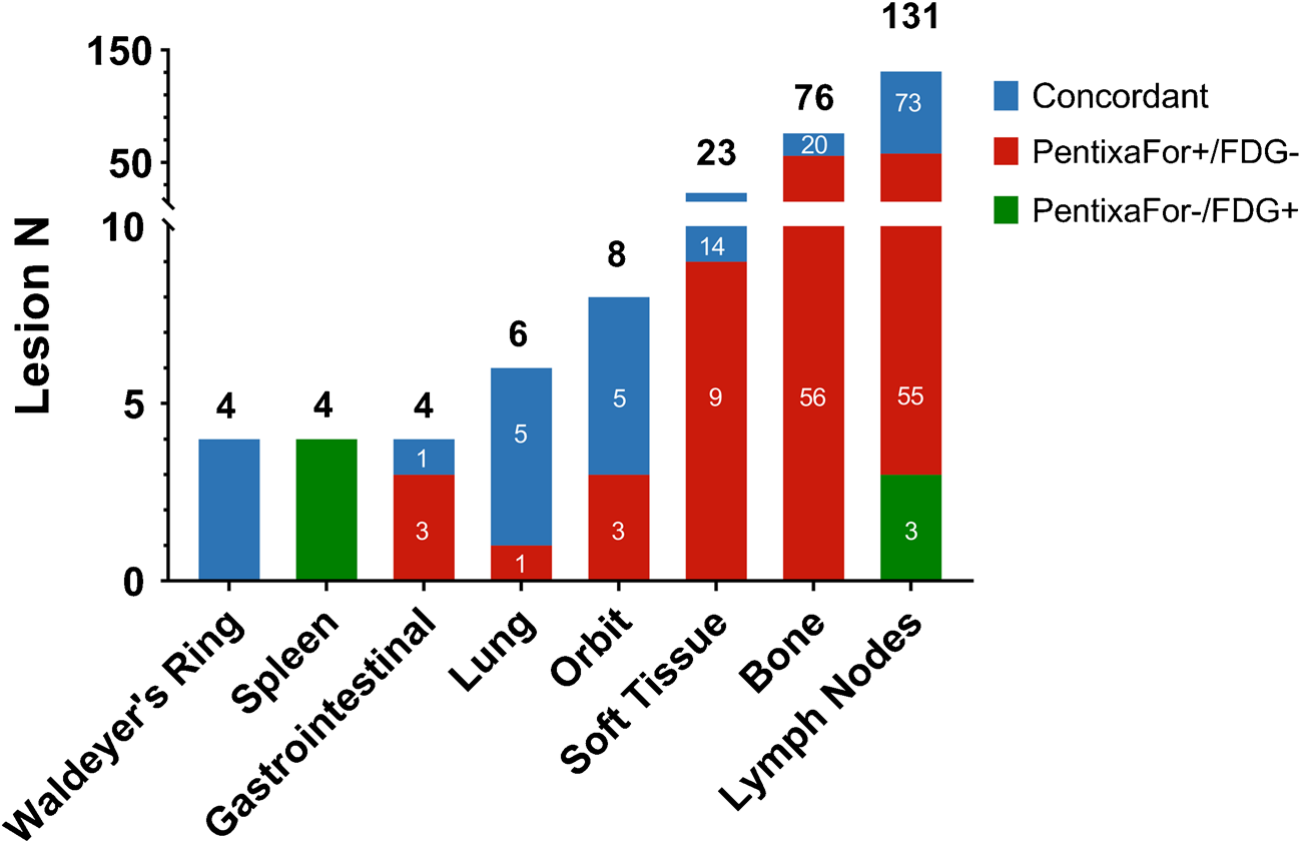
Bar graph showing numbers of marginal zone lymphoma lesions detected by [^68^Ga]Ga-PentixaFor PET/CT and [^18^F]FDG PET/CT per organ compartment

On a patient-based level, 32/32 (100%) were positive for MZL manifestations on [^68^Ga]Ga-PentixaFor, while only 24/32 (75.0%) patients were rated positive on [^18^F]FDG PET/CT. The 8/32 (25.0%) of scans which were only positive on [^68^Ga]Ga-PentixaFor, but missed by [^18^F]FDG PET/CT on a visual assessment had the following subtypes: EMZL 5/8 (62.5%), NMZL 2/8 (25.0%), followed by SMZL 1/8 (12.5%). A total of 256 lesions were identified (concordant on both scans, 122/256 (47.7%); [^68^Ga]Ga-PentixaFor+/[^18^F]FDG-127/256 (49.6%); [^68^Ga]Ga-PentixaFor-/[^18^F]FDG+, 7/256 (2.7%)). Fig. 1 and Table 1 provide a comprehensive overview of MZL lesions per organ system.

**Table 1:** Numbers of marginal zone lymphoma lesions per organ system detected by [^68^Ga]Ga-PentixaFor PET/CT and [^18^F]FDG PET/CT

Organ	Concordant (N)	[^68^Ga]Ga-PentixaFor+/[^18^F]FDG- (N)	[^68^Ga]Ga-PentixaFor-/[^18^F]FDG+ (N)	Sum (N)
Waldeyer’s tonsillar ring	4	0	0	4
Spleen	0	0	4	4
Gastrointestinal tract	1	3	0	4
Lung	5	1	0	6
Orbit	5	3	0	8
Soft tissue	14	9	0	23
Bone	20	56	0	76
Lymph node	73	55	3	131
Sum	122	127	7	256

### Concordant and discordant lesions on CXCR4-directed and [^18^F]FDG PET/CT

Concordant lesions on both scans exhibited significantly higher median SUV_max/peak_ on CXCR4-directed imaging vs. [^18^F]FDG PET/CT: SUV_max_, 10.3 (range, 2.52–37.2) vs. 5.72 (range, 2.32–37.0); SUV_peak_, 6.23 (range, 1.58–25.7) vs. 3.87 (range, 1.54–27.7); *P* < 0.01, each. Similarly, for concordant lesions, TBR on [^68^Ga]Ga-PentixaFor PET (median, 3.85; range, 1.05–16.0) was approximately 1.8-fold higher compared to [^18^F]FDG PET (median, 2.08; range, 0.81–28.8; *P* < 0.01).

**Fig. 2:**
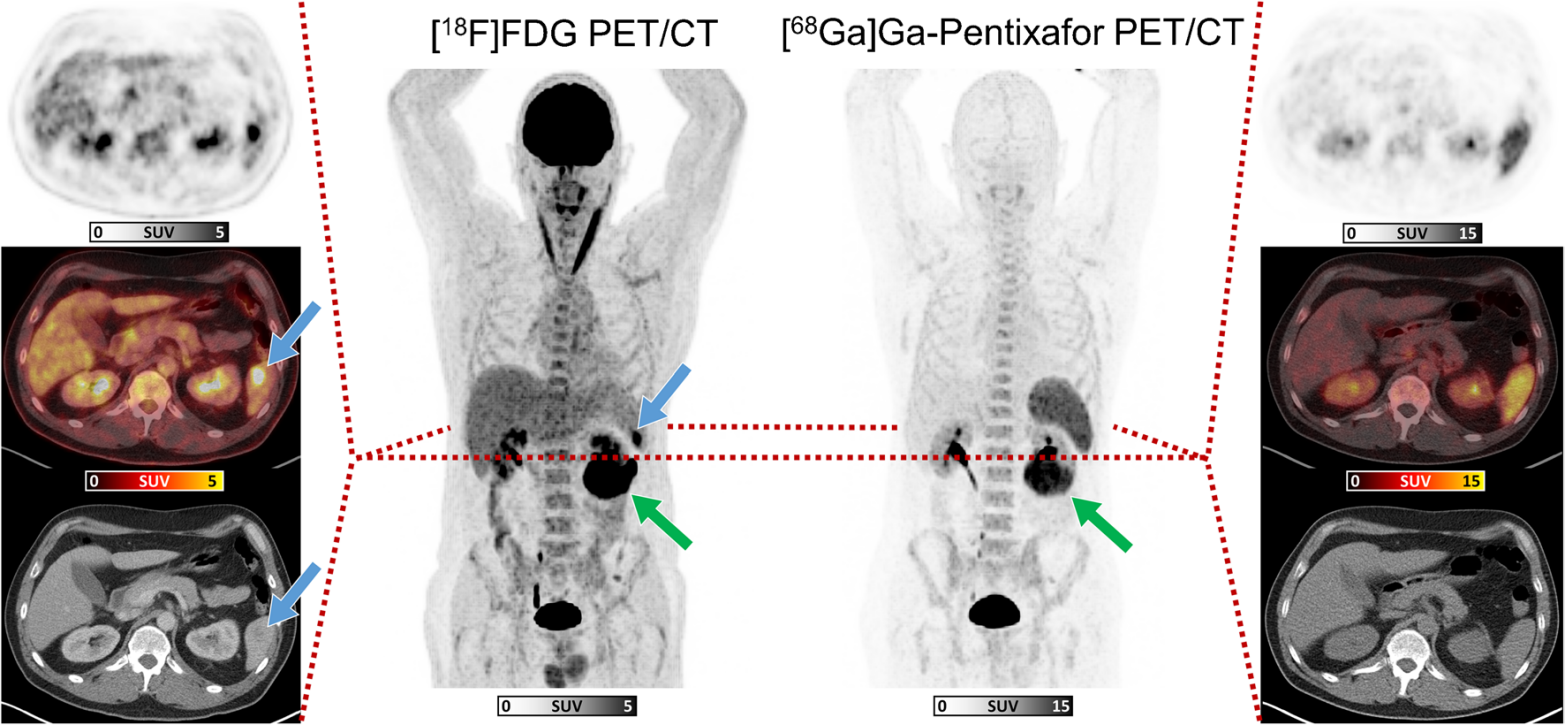
Maximum-intensity-projections (MIP) of [^18^F]FDG PET/CT (center left) and [^68^Ga]Ga-PentixaFor PET/CT (center right) in a 54-year-old male patient affected with extranodal marginal zone lymphoma, with axial sections ([^18^F]FDG: left column, and [^68^Ga]Ga-PentixaFor: right column) of PET (top row), fused image (middle row), and CT (lower row). Blue arrows indicate a splenic lesion, evident only on [^18^F]FDG PET/CT. Green arrows on MIPs indicate a renal lesion seen on both PET scans (not shown on axial sections)

For the discordant lesions, the following quantitative metrics were recorded. [^68^Ga]Ga-PentixaFor+/[^18^F]FDG-lesions showed an overall CXCR4-based SUV_max_ of median 7.74 (range, 3.13–25.8), and SUV_peak_ of median 4.48 (range, 1.89–19.3), with a median TBR of 2.54 (range, 1.09–9.80). The majority of [^68^Ga]Ga-PentixaFor+/[^18^F]FDG- MZL manifestations was recorded in lymph nodes, osseous structures, and soft tissue (Fig. 1). Of note, out of four gastrointestinal MZL manifestations, three (75.0%) were visualized solely on [^68^Ga]Ga-PentixaFor PET/CT. [^68^Ga]Ga-PentixaFor-/[^18^F]FDG+ lesions were noted in the spleen (Fig. 2) and in lymph nodes (Fig. 1), with an overall [^18^F]FDG-based SUV_max_ of median 9.74 (range, 5.41–25.1), and SUV_peak_ of median 6.91 (range, 3.85–19.6), with a median TBR of 4.27 (range, 1.45–9.31).

### Quantitative assessment of CXCR4-directed and [^18^F]FDG PET/CT per MZL subtype

Table 2 shows results of a lesion-wise analysis of quantitative PET parameters, grouped by MZL subtype. For both radiotracers, most MZL lesions were evident in NMZL, followed by EMZL and SMZL. However, [^68^Ga]Ga-PentixaFor PET/CT consistently showed substantially more MZL lesions relative to [^18^F]FDG PET/CT, regardless of MZL subtype ([^68^Ga]Ga-PentixaFor vs [^18^F]FDG: NMZL: *N* = 216 vs *N* = 114; EMZL: *N* = 17 vs *N* = 13; SMZL: *N* = 16 vs *N* = 2).

**Table 2:** Quantitative analyses of marginal zone lymphoma (MZL) lesions presented by subtype for [^68^Ga]Ga-PentixaFor and [^18^F]FDG PET

		NMZL			EMZL			SMZL*	
		[^68^Ga]Ga-PentixaFor	[^18^F]FDG	P	[^68^Ga]Ga-PentixaFor	[^18^F]FDG	P	[^68^Ga]Ga-PentixaFor	[^18^F]FDG
Overall	N	216	114		17	13		16	2
	SUV_max_	8.69 (3.53–37.2)	5.72 (2.32–37.0)	**< .01**	9.75 (2.53–25.1)	7.19 (3.94–17.7)	0.17	7.36 (5.52–10.1)	3.00 (2.49–3.50)
	SUV_peak_	4.99 (1.81–25.7)	3.84 (1.54–27.7)	**< .01**	4.82 (1.58–16.5)	5.36 (2.88–14.3)	0.67	4.44 (2.94–6.93)	2.18 (1.69–2.67)
	TBR	3.13 (1.14–16.0)	2.08 (0.82–28.8)	**< .01**	4.06 (1.05–8.66)	2.47 (1.58–8.85)	0.34	2.50 (1.65–4.13)	1.05 (0.81–1.28)
Concordant	N	108	108		12	12		2	2
	SUV_max_	10.2 (3.53–37.2)	5.51 (2.32–37.0)	**< .01**	14.5 (2.53–25.1)	7.09 (3.94–17.7)	**0.04**	6.56 (5.52–7.59)	3.00 (2.49–3.50)
	SUV_peak_	6.14 (1.81–25.7)	3.74 (1.54–27.7)	**< .01**	10.5 (1.58–16.5)	5.21 (2.88–14.3)	**0.04**	3.91 (2.94–4.87)	2.18 (1.69–2.67)
	TBR	3.79 (1.29–16.0)	2.07 (0.82–28.8)	**< .01**	5.18 (1.05–8.66)	2.40 (2.16–11.0)	0.06	2.20 (1.65–2.74)	1.05 (0.81–1.28)

Numbers indicate median with range in parenthesis

*NMZL* nodal marginal zone lymphoma, *EMZL* extranodal marginal zone lymphoma, *SMZL* splenic marginal zone lymphoma, *SUV* standardized uptake value, *TBR* target to background ratio

Significant *p*-values are set in bold. *p*-values were considered significant if *p* was less than 0.05

^*^Due to the small number of lesions on [^18^F]FDG PET/CT in SMZL (*N* = 2), a reliable indication of a *P* value is not possible

For NMZL, CXCR4-directed imaging showed significantly higher SUV_max/peak_ and TBR for all visible MZL and concordant lesions when compared to [^18^F]FDG PET/CT (*P* ≤ 0.01; Table 2). In patients with EMZL, there was no significant difference comparing all lesions detected by both radiotracers (*P* ≥0.17); however, considering concordant MZL lesions only, [^68^Ga]Ga-PentixaFor PET/CT showed significantly higher SUV_max/peak_ (*P* = 0.04), but just missed significance for TBR (*P* = 0.06; Table 2). Due to the small number of MZL lesions on [^18^F]FDG PET/CT in SMZL (*N* = 2), no such quantitative head-to-head comparison has been conducted.

## Discussion

In the present visual and quantitative analysis of the total lymphoma load on [^18^F]FDG and [^68^Ga]Ga-PentixaFor PET/CT, we observed that the latter radiotracer revealed MZL manifestations in a higher proportion of patients, along with substantially increased detection rate on a TL level. In a read-out of quantitative PET parameters for concordant lesions on both scans, [^68^Ga]Ga-PentixaFor almost consistently provided significantly higher SUV_max_, SUV_peak_, and TBR, indicative for improved contrast. In a subgroup analysis of MZL subtypes, visual and quantitative comparison of both radiotracers also provided higher diagnostic performance of [^68^Ga]Ga-PentixaFor PET/CT for NMZL and EMZL. As such, based on our visual and quantitative comparison, the latter PET agent may emerge as the radiotracer of choice when investigating MZL patients upon initial diagnostic work-up.

As the reference radiotracer in nuclear oncology, [^18^F]FDG is well-established in the vast majority of lymphomas, in particular for treatment monitoring [15]. Not surprisingly, this agent has also been extensively validated in patients with MZL, but its diagnostic accuracy seems to vary based on the investigated subtype. For instance, Hofmann et al. reported that [^18^F]FDG PET is positive in nodal, but not EMZL, which is in line with our findings reporting on limited detection rate for the latter subtype on a TL level (Table 2) [16]. Nonetheless, the clinical value of this radiotracer remains a matter of debate, as a recent study investigating all three MZL subtypes reported on consistent upstaging after [^18^F]FDG administration when compared to CT alone [17]. Given those controversial findings, novel radiotracers providing a more reliable read-out of the current disease extent are intensively sought. In this regard, theranostic radiotracers may provide improved staging along with potential therapeutic options using ß-emitting 177Lu-labelled counterparts. Recent immunohistochemical analysis, however, revealed limited or even abundant expression of somatostatin receptors (SSTR) 2a/5 [18, 19], which are specifically addressed by SSTR-directed radiotracers used for imaging and therapy [20]. Not surprisingly, in vivo targeting of this receptor subtype then provided a substantial rate of false-negative findings, thereby rendering SSTR as less relevant in the context of MZL [21]. CXCR4, however, was substantially upregulated in more than 90% of samples obtained from extranodal MZL of mucosa-associated lymphoid tissue lymphoma [18]. Also exploited as theranostic target, such chemokine receptors have been subject to multiple imaging studies using [^68^Ga]Ga-PentixaFor [10–12]. For instance, a recent analysis focusing on the diagnostic performance of CXCR4-directed PET/CT relative to guideline-compatible diagnostic work-up (CT, bone marrow biopsy and esophagogastroduodenoscopy) also provided preliminary evidence on the superiority of this radiotracer relative to [^18^F]FDG. However, in this previous study, only one single TL, defined as most intense on uptake, was analyzed [10]. In the present investigation, we expanded those preceding findings by examining the entire whole-body lymphoma burden on a visual and quantitative level, which then also provided a comprehensive compartment- and subtype-based analyses. Of note, for both lymphonodal and extranodal compartments, CXCR4-targeted imaging revealed substantially more sites of disease which would have been missed by [^18^F]FDG, in particular in manifestations located in the soft tissue and bone. Although limited by the retrospective nature and small number of enrolled subjects, our study may provide a hint that [^68^Ga]Ga-PentixaFor may emerge as the novel diagnostic PET agent of choice in MZL. These considerations are also further fueled by an observed elevated TBR in both NMZL and EMZL. Indicative for improved image contrast, those findings suggest that CXCR4-directed imaging may address the clinical need of a reliable diagnostic PET agent, in particular in EMZL, where [^18^F]FDG only provides rather limited clinical benefit. In this regard, future studies should determine whether the observed high image contrast on [^68^Ga]Ga-PentixaFor PET indeed improve reader’s confidence, e.g., by conducting an interobserver agreement study [22]. Nonetheless, chemokine receptor imaging may be less suitable in patients with splenic involvement, which is partially explained by the physiological biodistribution of this agent [23].

Beyond image contrast, we also recorded SUV_max_ of median >10, with individuals even exhibiting values above 37. Based on those PET metrics, therapeutic applications using [^177^Lu]Lu- or [^90^Y]Y-PentixaTher would then provide anti-lymphoma efficacy, along with (desired) eradication of the stem cell niche as an integral component of the treatment plan, e.g., to prepare for subsequent stem cell transplantation [24]. As such, relative to [^18^F]FDG, the theranostic aspect of CXCR4-targeted imaging would then not only allow for better delineation of putative sites of disease, but also identify patients eligible for treatment.

Last, several limitations of this study should be noted. First, this study’s retrospective nature implies, that it is not possible to histologically confirm specific lesions seen on molecular imaging. In addition, not all lesions that were conspicuous on imaging can be biopsied and confirmed histologically. However, the fact that several readers verified inconclusive lesions in consensus should contribute to diagnostic confidence. Moreover, previous work has shown that CXCR4-directed imaging can be used as an accurate staging tool in patients with marginal zone lymphoma [10]. In this regard, Duell and colleagues [10] demonstrated that 16 out of 18 PET-guided biopsies of suspicious lesions exclusively visualized on [^68^Ga]Ga-PentixaFor PET compared to [^18^F]FDG were actually histologically confirmed as MZL manifestations. Therefore, even lacking bioptic confirmation of each lesion documented on imaging, we believe that viewed with due caution, our results are of value.

## Conclusions

Compared to [^18^F]FDG, [^68^Ga]Ga-PentixaFor PET/CT identified more sites of disease in a higher proportion of patients with newly diagnosed MZL, irrespective of the investigated subtype. PET quantification — including TBR — provided consistently higher values on [^68^Ga]Ga-PentixaFor PET, thereby also suggesting improved image contrast. As such, in patients affected with MZL, our findings may pave the way for a more widespread adoption of this PET agent in the clinic.

## Data Availability

The datasets generated and analyzed during the current study are available from the corresponding author upon reasonable request.
